# Immediate and Persisting Effects of Controversial Media Information on Young People’s Judgement of Health Issues

**DOI:** 10.5964/ejop.v16i2.1929

**Published:** 2020-05-29

**Authors:** Attila Szabo

**Affiliations:** a Institute of Health Promotion and Sport Sciences, ELTE Eötvös Loránd University, Budapest, Hungary; b Institute of Psychology, ELTE Eötvös Loránd University, Budapest, Hungary; Glasgow Caledonian University, Glasgow, United Kingdom

**Keywords:** beliefs, influence, opinions, schema theory, views

## Abstract

Whether true or false, media information shapes people’s thinking. False information trigger beliefs which could compromise health behaviour. In this intervention study, the effect of controversial messages on 91 young participants’ judgement of health issues was tested. Held opinions about health-related issues were assessed before, after and one week after viewing a controversial TV-interview. Using pre-interview opinions for baseline, changes in judgements were assessed immediately after and one week after the interview. At both times, the opinions differed statistically significantly from baseline (p < .001). The relationship between opinions immediately- and one week-after viewing the interview was statistically significantly stronger (p < .001) than their association with the baseline. The results provide evidence for immediate change in judgment resulting from controversial information and demonstrate that the change persists for at least one week. The findings can be explained based on the schema theory and suggest that controversial information could have powerful impact on subjective judgement. Consequently, young people need to be educated in health issues and in the evaluation of media information to enable them to make the right choice when the need arises.

Media can prime people’s opinion (Roskos-Ewoldsen, Klinger, & Roskos-Ewoldsen, 2007). There are several theories for the media effects on people. Three popular models are the agenda-setting, priming, and framing (Scheufele & Tewksbury, 2007). Agenda-setting is a model which purports that there is a strong relationship between the emphasis (placement and amount, order and priority, and frequency and duration) of certain issues in the mass media and the subjective importance attributed to these topics by their audience (McCombs & Shaw, 2017). Priming is the influence on people’s judgement that changes the standards individuals use to evaluate certain issues (Iyengar & Kinder, 1987). Framing reflects the directed perspective in which a subject matter is presented in the media that can influence its interpretation by the audience (Scheufele & Tewksbury, 2007). Agenda-setting and priming are *accessibility* effects requiring attention to an issue to be stored in the memory (Price & Tewksbury, 1997). In contrast, framing pertains to the description or meaning of an issue. The main difference between agenda-setting and priming in contrast to framing is in attention (accessibility) and outcome effect (applicability), which is driven by interpretation, or information processing (Price & Tewksbury, 1997). However, applicability also requires accessibility, otherwise it could not affect the judgment of the person.

From a psychological perspective, the attended information first is stored in the short-term memory and via repeated, or a high impact, exposure (accessibility) and deeper processing (applicability) is stored into the long term the memory (Scheufele & Tewksbury, 2007). In accord with the *Schema theory* (Axelrod, 1973), the stored information form schemas or blueprints in the memory to be retrieved later. The accessing of a schema for new information is sequential. Highly accessible schemas are processed first whereas less accessible schemas are processed last in the attempt to find a schema that fits the new information. If a schema is found, then the new information is evaluated against this schema. In the case of a close match between the existing schema and the new information, the old schema is updated accordingly. However, if the new information does not fit well the old schema, the information is subjected to cognitive judgement (credibility, doubt) whereby it is either refuted or is combined with the old schema to yield a modified schema. According to the theory, the newly generated schema emerges with new specifications with upgraded accessibility, credibility, and confidence in its interpretation (Axelrod, 1973, Figure 1, p. 1251). The credibility of the source is an important determinant in the schema classification of the new information (Greer, 2003). Based on the schema theory, highly credible and high impact information will form enduring schemas, but the less credible ones may either dissipate or form short-enduring quickly reformed schemas.

The effects of media information on individuals’ judgements are generally studied in media-priming research (Roskos-Ewoldsen, Roskos-Ewoldsen, & Dillman Carpentier, 2002). Initially, priming was used in cognitive psychology to study how information is represented within the network models of memory (Anderson, 1996). Different schools of thought assign seemingly distinct meaning to priming, but they agree that priming involves acute exposure to media information that influences people’s feelings, judgements and behaviours (Iyengar & Kinder, 1987). In the network models of memory (Anderson, 1996) the information is stored in form of nodes. Each node symbolizes a concept (e.g., health). The nodes are closely linked to related nodes (i.e., health is linked more closely to *‘fitness’* than to *‘inflation’*). Each node has an activation threshold. If activation reaches the threshold, the node fires while it exerts a strong influence on the activation of the associated nodes (i.e., if the ‘*fitness*’ node fires then the activation may spread to the ‘*health*’ node). This model is in accord with the schema theory (Axelrod, 1973), but the latter emphasizes more applicability in schema transformation than the primarily accessibility-oriented network model. Information priming activates nodes aiding in schema retrieval for evaluation and, if necessary, restructuring.

Conceptually, priming may be closely related to the nodes in the memory network aiding in their activation. Its effects are often subject to relatively short time decay as it was revealed in past research (Arendt, 2013; Dillman Carpentier, Roskos-Ewoldsen, & Roskos-Ewoldsen, 2008; Riddle, 2010; Sloman, Hayman, Ohta, Law, & Tulving, 1988). In contrast, framing may be more closely related to cognitive schemas (Entman, Matthes, & Pellicano, 2009). Its effect may last longer (Druckman & Nelson, 2003; Lecheler & de Vreese, 2011; Tewksbury, Jones, Peske, Raymond, & Vig, 2000). Despite past studies demonstrating the impact of framing on people’s judgement (Gunter, McAleer, & Clifford, 1991; Taylor, 2005), its decay over time is not clarified in the literature. In a political context, Lecheler and de Vreese (2011) found that the effects of framing are relatively persistent, however they are mediated by the prior knowledge of the individual; moderate knowledge is associated with the most persistent framing effects, while high or low political knowledge is linked to faster dissipating effects. Based on the link between prior knowledge and framing effect, these findings agree, but also disagree, with the earlier results obtained by Schuck and de Vreese (2006), who showed that the framing effect was moderated by political knowledge of the person, but those with little political knowledge were more affected by framing. Thus, the impact and duration of framed information appears to be moderated by the relevant knowledge of the individual.

Framing research is primarily focused on political- and violence issues. Empirical work on the framing of health information is relatively meagre. Abundant controversial information exists, in various media, about nutritional supplements, slimming products, pain-reliefers, and other similar products to motivate their consumption (Philen, Ortiz, Auerbach, & Falk, 1992). Studies looking at the framing of health information often measure behaviour (consumption) as the outcome variable. Unexpectedly, in contrast to political and violence framing, health-oriented framing appears to have little or no clear effect on health consumer behaviour according to a Cochrane systematic review (Akl et al., 2011). Further, negatively framed messages appear to be more effective than positively framed messages, but both are prone to time decay (Rivers, Salovey, Pizarro, Pizarro, & Schneider, 2005). Taken together, the extant literature suggests that framed messages affect people’s judgement, but their effect is mediated by many factors and their impact on health behaviours is unclear. Framed information appears to be prone to time decay.

The current study investigated how effects of controversial information, framed through credible media (public television) and presented as originating from a credible source, could instantly alter young people’s judgement and whether the altered judgement persists one week later. In contrast to many past inquiries, this study did not rely on volunteers, its content was health-related, but not associated with health-consumerism, and accounted for participants’ existing knowledge and convictions by using a baseline to which the magnitude of change, solely due to the intervention, could be ascribed. Two hypotheses were tested: 1) Despite very controversial information, young people will change their mind about three very actual health/medical issues: medication, vaccination and medical doctors’ reliability, immediately after exposure to the information, and 2) the change in judgement (if evident) will prevail for at least one week. In fact, it was conjectured that the framed information will modify the existing schema (at baseline) of the participants and this new altered schema will be rather permanent and, therefore, detectable one week later as well.

## Method

### Participants

The study was presented within the curriculum of a second-year research methods course at ELTE Eötvös Loránd University. This method was used to eliminate bias due to voluntary participation (Rosenthal, 1965). Ethical approval for the current research, which was embedded into the study-curriculum, was granted by the Research Ethics Committee of the Faculty of Education and Psychology at ELTE Eötvös Loránd University in Budapest, Hungary. The sample consisted of 91 participants, 42 men and 49 women. Sample size adequacy was verified by using the G*Power (v. 3.1) software (Faul, Erdfelder, Buchner, & Lang, 2009) with the following input parameters: alpha (α) = .05, effect size (Cohen’s *d*) = 0.5, power (I – β) = .95. The results of the test yielded a minimum required sample size of 44 for paired t-tests and 88 for independent tests.

Participants’ mean (*M*) age was 21.18 ± *SD* = 1.47 years. They were sport science students who received no prior education in psychology. Although examining university students, particularly psychology students, in research has been criticized (Henrich, Heine, & Norenzayan, 2010), this age group is especially vulnerable to media information (Hargittai, Fullerton, Menchen-Trevino, & Thomas, 2010). Further, young people’s judgement of health issues may be frail, because - in general – they have fewer health concerns than older adults (Dieleman et al., 2016). The study was totally anonymous. Apart from age and gender, participants did not provide any other personal information through which they could be identified.

### Materials

A 24-minute media-recorded television interview, which is available on the internet, with a famous retired cardiac surgeon having strong and controversial opinions, such as “*vaccination should be obligatory and regulated by law*” or “*cholesterol-lowering medication can kill*” was presented to sport science students attending a regular class. None of them was familiar with this interview. On three different occasions, they rated either with ‘*yes*’ (agree) or ‘*no*’ (disagree) six statements, five of which reflected the views of the cardiac surgeon giving the interview: 1) “*This nation is addicted to medication.”,* 2) *“The doctors make deadly mistakes and often mislead us.”*, 3*) “Cholesterol lowering drugs have the opposite effect and can kill.”,* 4) *“The parent who does not vaccinate her child could be considered a murderer.”,* 5) “*The vaccine against the influenza virus contains poisonous mercury.”*, and 6) *“Professor (name) is a very famous cardiac surgeon with international reputation.”* The statements were taken directly from the recorded interview. The first five statements were made by the interviewee and the last by the interviewer. The whole interview revolved around the first five questions.

### Procedure

#### Research Venue and Passive Consent for Participation

The class entitled “being participant in psychology research” was planned for the second class of the semester in a large lecture hall. It was scheduled for the late morning hours. Although, the current study was part of the course curriculum, passive consent was obtained by projecting a consent form on a large screen which clearly highlighted that by providing answers, which are totally anonymous, the students consent to participation. This method is identical to consent obtained in online studies (British Psychological Society, 2017). Consequently, there was no pressure to participate. However, none of the class attendees declined participation.

#### Information Provided to the Participants

The students were informed that the level of their convictions concerning certain health and medical issues is measured at three different times to determine the stability of the personal beliefs over time. Then they were told that after assessing their convictions at baseline (the very first measurement before the presentation of the video-interview) they will watch a video that could distract their convictions. The deception concerning the stability of personal beliefs as the primary goal of the study was necessary to minimize response bias and it was disclosed and clarified upon the termination of the study. (In fact, all the results and all possible interpretations were discussed as part of the lecture material even with those who were absent at the time(s) of data collection.)

#### Data Collection

All participants received three pieces of identically coded answer sheets. The codes were identical to ensure the later matching of the responses. The distribution and collection of the answer sheets, as well as all the projections on the classroom screen, were performed by an older student research assistant in the absence of the class instructor. On the first answer sheet (baseline measure), participants indicated their age and gender (no other personal information was collected) and then either agreed with a ‘*yes*’ or disagreed with ‘a *no*’ to the six statements described in the Materials section. (These answer sheets did not contain the statements, which were projected on a large classroom screen, but simply the *yes* or *no* answers preceded by the number of the statement.) Immediately before the video, the student research assistant, who also started and stopped the video, collected the first set of completed answer sheets, while the other two were kept by the participants for later.

#### The Projected Video-Interview

Before starting the video, the students were asked to pay full attention to the interview. The video was played on large screen between 70-75 decibel (dB) in a large classroom and it started with the interviewer’s introduction of the interviewee of the documentary as a world-renowned cardiac surgeon. This introduction stressed the credibility of the interviewee. Once the interview ended, participants were asked to complete the second answer sheet containing the same six statements as the one completed before the intervention. Subsequently, the student research assistant collected the answer sheets and participants were told to keep the third answer sheet for the following week.

#### Assessing Residual Effects and Debriefing

One week later, immediately before the start of the lecture, participants completed the third answer sheet while the six statements (no video at this time) were projected on the class screen. After the collection of the answer sheets by the student research assistant, the lecturer entered the class and explained the purpose of the study to the students while their questions were answered. After completing the data analyses, the results of the study and their possible implications were collectively discussed as part of the lecture material.

### Data Analyses

Paper and pencil data were manually entered in an Excel file, verified for accuracy, and imported into SPSS data-file. Since 15 participants were absent one week after the presented video interview, the third data collection resulted in a lower sample size (*n* = 76) in contrast to the first and second data collection which took place on the same day. The sum of the ‘yes’ answers were computed for each case for each sampling occasion. Next, the sum obtained before the interview (the baseline) was subtracted from the sum obtained immediately after- and one week after the video watching. This calculation resulted in two ‘change-scores’ also known as the ‘delta (Δ) scores’; one accounting for the difference between the baseline and immediately after the video (*n* = 91) and one between the baseline and one week after the video (*n* = 76). The change-scores were subjected to one sample t-test, using a reference value *M* = 0, testing the null hypothesis that the observed change equals to zero. The two sets of change-scores were also compared to each other with a paired t-test to examine whether their magnitude differed at the two sampling occasions. When a statistically significant difference was found, the effect size (Cohen’s *d*) was computed too. Bootstrapping (1000 samples) was used in all tests to gather more accurate confidence intervals (Banjanovic & Osborne, 2016). Correlations between the sum of the ‘yes’ answers, indicating agreement with the presented statements, were calculated to examine the congruity between the personal opinions at the three sampling times.

## Results

**Figure 1:**
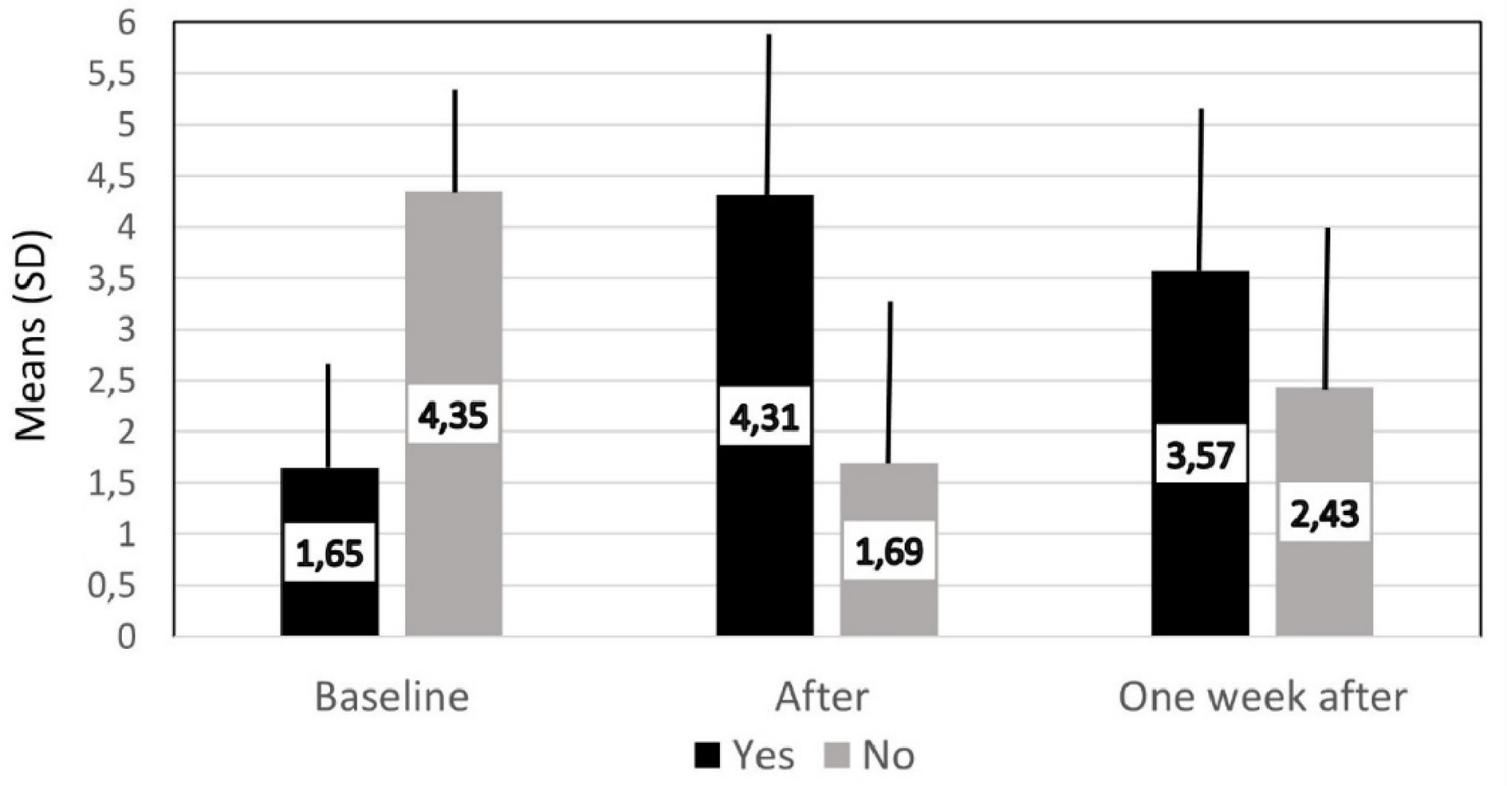
Mean number of ‘yes’ (reflecting agreement) and ‘no’ (reflecting disagreement) answers at baseline, immediately after- and one week after the intervention.

**Figure 2:**
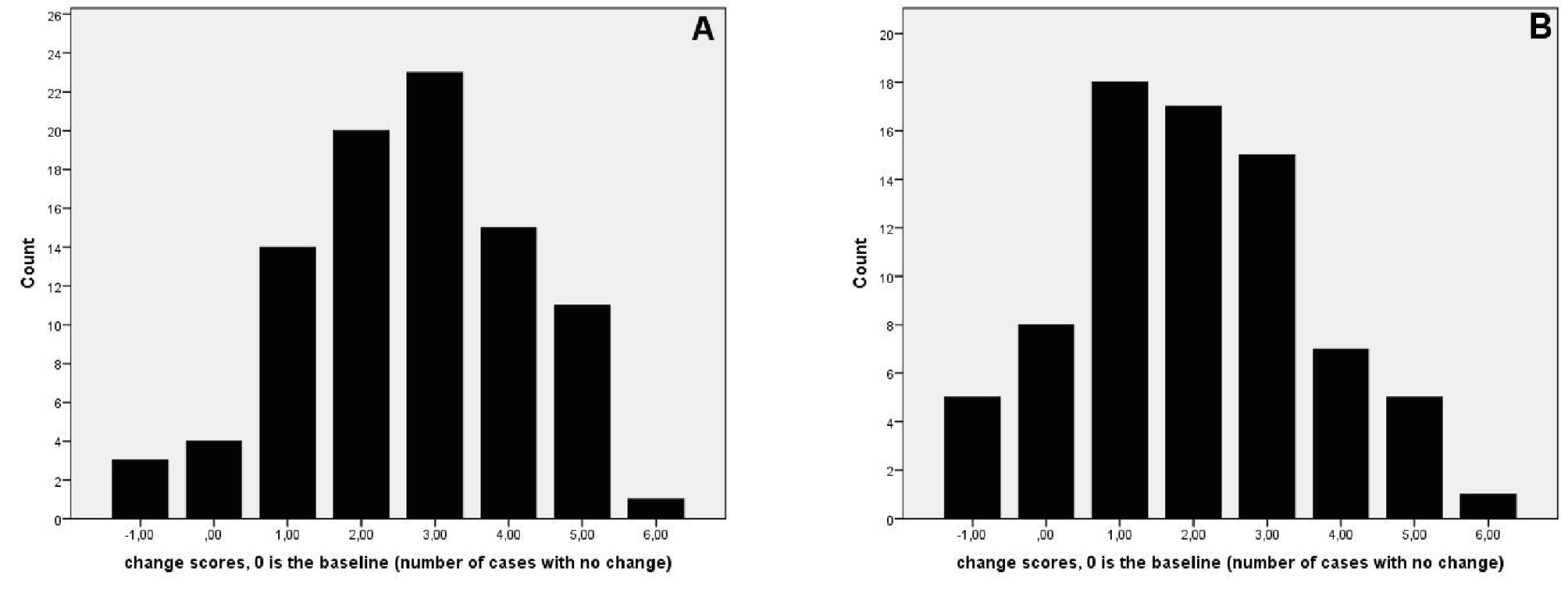
Number of changes in opinions on six statements immediately after (A) and one week after (B) the video interview intervention. The range of possible change scores is -6 to +6. Changes in the negative direction indicate changes towards disagreement and changes in positive direction reflect changes towards agreement. Cases at 0 (.00), four immediately after and one week after, reflect no-changes in opinions on the six statements in comparison to the baseline. A +6 (one case in both figures) reflects change in opinion (from disagree to agree) on all six statements.

The sum of agreements (yes) and disagreements (no) before and two times after watching the interview are illustrated in Figure 1. The number of changes out of six possible, immediately after the interview and one week after the interview are illustrated in Figure 2A and Figure 2B, respectively. An initial independent *t*-test, testing whether there were gender differences in the two sets of change scores, yielded statistically no significant results, *p* > .05. Subsequently, the data obtained from the two genders were examined together.

The bootstrapped one sample *t*-test examining whether change scores (or difference scores between the ‘yes’ answers; see Figure 1) obtained immediately after the video differed from zero was statistically significant and yielded a large effect size (Table 1). In fact, only four respondents did now show a change in any of their six existing (baseline) opinions after the interview. Another *t*-test, examining the change scores one week after the interview, was statistically significant again and yielded a large effect size (Table 2). The mean values of the change scores immediately following- and one week after the video watching also differed statistically significantly from each other, but only yielded a moderate effect size (Table 3). An important finding is that 36 (47.4%) participants *did not change* again their opinion on any of the six statements within one week. Out of these 36 individuals, only two did not change their judgement from baseline to immediately after the video assessment. This means that 34 of the participants from among who changed their opinions from baseline to immediately after intervention (all but four) fully maintained their new opinions one week later as well.

**Table 1:** Results of the One-Sample T-Test Testing the Change in Opinions From Baseline to Immediately After Watching the Video

Bootstrap one sample *t*-test (based on 1000 samples)	
*M*, *SD*	2.6484 (1.5519)
Bias (*M*, *SD*)	-.0134 (-.0117)
Standard error (*M*, *SD*)	.1591 (.1037)
95% Confidence interval	2.3077 (1.3293) to 2.9451 (1.7616)
Range	-1 to 6
*t* (*df*)	16.279 (90)
*p* (2-tailed) and effect size (*d*)	.001, 1.71

*Note. M* = mean; *SD* = standard deviation; *df* = degrees of freedom; *d* = effect size (Cohen’s *d*).

**Table 2:** Results of the One-Sample T-Test Testing the Change in Opinions From Baseline to One-Week After Watching the Video

Bootstrap one sample *t*-test (based on 1000 samples)	
*M*, *SD*	1.9868 (1.6289)
Bias (*M*, *SD*)	-.0064 (-.0175)
Standard error (*M*, *SD*)	.1836 (.1199)
95% Confidence interval	1.6316 (1.3731) to 2.3553 (1.8354)
Range	-1 to 6
*t* (*df*)	10.634 (75)
*p* (2-tailed) and effect size (*d*)	.001, 1.22

*Note. M* = mean; *SD* = standard deviation; *df* = degrees of freedom; *d* = effect size (Cohen’s *d*).

**Table 3:** Results of the Paired T-Test Testing the Change in Opinions From Immediately After Watching the Video to One-Week After Watching the Video (Decay Effects Within One Week)

Bootstrap paired *t*-test (based on 1000 samples)		
Statistical Indices	Immediately after watching the video	One week after watching the video
*M*, *SD*	2.6316 (1.5819)	1.9868 (1.6289)
Bias (*M*, *SD*)	-.0176 (-.0143)	.0135 (-.0095)
Standard error (*M*, *SD*)	.1804 (.1235)	.1935 (.1107)
95% Confidence interval	2.3026 (1.3300) to 2.9868 (1.7992)	1.6447 (1.3931) to 2.3816 (1.8431)
Mean difference (*SD*)	.6447 (1.1512)	
*t* (*df*)	4.882 (75)	
Bias (Standard error)	.0040 (.1305)	
*p*	.001	
Effect size (*d*)	.56	
95% Confidence interval	.3819 to .9207	

*Note. M* = mean; *SD* = standard deviation; *df* = degrees of freedom; *d* = effect size (Cohen’s *d*).

Correlation analyses indicated that the number of ‘yes’ answers given immediately after- and one week after the biased video interview correlated statistically significantly, but relatively weakly, with pre-video watching personal opinions, or ‘yes’ answers, *r* = 354, *r*^2^ = .125, *p* = .001, and *r* = 348, *r*^2^ = .121, *p* = .002, respectively. However, a stronger correlation emerged for the number of yeses immediately after and one week after the intervention, *r* = 755, *r*^2^ = .570, *p* < .001. Based on Steiger’s (1980) calculation of the difference between two dependent correlations, the strength of the correlation between the two post-intervention ‘yes’ answers differed statistically significantly from the strength of the correlation found between pre-intervention and immediately post intervention, as well as from the correlation coefficient reflecting the association between pre-intervention and one week after the video watching, *z* = 3.513, *p* < .001, effect size (Cohen’s *d*) = 0.88 and *z* = 3.567, *p* < .001, *d* = 0.90, respectively.

## Discussion

Although presenting highly controversial information, the watching of a recorded television interview produced large changes in the evaluation of three health related issues (medication, vaccination, and doctors’ reliability) immediately after the intervention. The nearly three-fold change in agreement with questionable and controversial statements, was reflected in a very large effect size, which points toward an acute change in at least three related, but not identical schemas. These results fully agree with Axelrod’s (1973) schema theory. Participants’ schemas on health issues, influenced by controversial messages, were changed. The change, according to Axelrod, occurs as the result of a cognitive evaluation of the source- and information credibility versus an existing schema. This evaluation, as it may be expected, varies between the participants, because of individual differences in the strength of the respective schemas (Schmidt, 1975). Such variability is evident in Figure 2A and Figure 2B. An important finding may be that nearly half (47%) of the participants maintained their new opinions on all six statements one week after seeing the interview. A permanency in the new distorted schema, therefore, can by observed in nearly half of the participants. However, this observation should be treated carefully since a one-week persistence – although showing stability in several schemas - does not mean permanency. Indeed, two or three weeks later the re-assessment of the opinions might have resulted in different outcome. Therefore, to address the durability of the altered schemas longitudinal studies are necessary.

At the baseline, which represented the existing schema, there was about one quarter of agreement with controversial health statements, but after the presentation of the interview this figure almost tripled and it was still twice as much one week later (refer to Figure 1). Despite persistence of the effects in about half of the participants, the mean change from baseline was weaker one week later than immediately after the controversial video, however the effect size was only medium. These findings agree with earlier results showing that framing effects persist for a while, but generally they weaken over time (Druckman & Nelson, 2003; Lecheler & de Vreese, 2011; Tewksbury et al., 2000). It was suggested, that framing effects weaken in a relatively short period because there is no exposure to the framed information in that period (de Vreese, 2004). Therefore, a fading in the new, in this case a distorted, schema is likely the result of lack of use or reinforcement of the relevant schema, which was shown objectively in a motor schema research (Carson & Wiegand, 1979). On one hand, based on the schema theory it could be expected that participants without an existing schema for a health (or any other) issue develop a new information-based schema, which without reinforcement is weak and only persists until challenged or until is lost in the memory. On the other hand, people with an existing schema may alter/change that schema, but in lack of reinforcement they revert to the original schema over time. This renders research in the area rather complicated because qualitative studies may be needed to determine the existence and form of a schema concerning a subject matter. Subsequently, a quantitative approach should be adopted to investigate the magnitude and duration of the change in existing and non-existing (i.e., not-yet-existing or newly formed) schemas over time in longitudinal investigation.

Another noteworthy result of the current research is the stronger correlation between the two post-intervention agreements (i.e. the ‘yes’ answers sharing 57% of the variance) vis-à-vis their individual correlations with the pre-intervention baseline (sharing close to 35% of the variance in both cases). The statistically significant differences between the two correlations suggests that the schemas after the intervention were more closely associated with each other than with the pre-existing schema. This difference in the strength of association supports both a shift in the original schemas and a greater congruity between the shifted/altered schemas in contrast their congruity with the original schemas. No research could be located testing the association between framed effects compared to a baseline, despite such research may provide a better overall picture about the impact of a framing intervention.

### Practical Implication

The current findings demonstrate how exposure to controversial and possibly distorted information about health issues in the media could largely affect young people’s subsequent opinion or judgement within a few minutes. The age-group studied in this work is the *largest* Internet consumer (Eurostat, 2016), which along with the television represent the two most popular media (Standard Eurobarometer 82, 2014). The large effect sizes associated with the current results reveal that there is a large change in the judgement attributable to the information from the media, whether that information is real or questionable. Such findings should attract attention to the dangers of biased, false and/or misleading information, which could be – without exaggeration (see Khomami, 2015) – even deadly. Beyond research, the internet-transformed societies’ primer goal should be the education of young people to defend themselves from the false, distorted and dangerous information from the media, especially the internet, by showing them how to separate fact from opinion (Austin & Pinkleton, 2016). Future studies need to investigate further and expand the current results in the context of age-groups, culture, education level and/or socio-economic status, and personality traits.

### Strengths and Limitations

The strength of this research is the use of a non-volunteer sample, as volunteerism results in biased results (Rosenthal, 1965). Another strength of the study is that it focused on several (six) pieces of information and, therefore, multiple schemas, which possibly contributed to more reliable findings than focusing on a single schema when considering changes in judgements. Another possible strength is the adoption of a baseline to which change scores were compared. The latter may account for the between-subject variability in the strength of the schemas and yield quantitative data that are attributable to the intervention. However, the study has several limitations too. First, it did not measure level of confidence in the viewed interview, which is known to be a mediating factor in framing effects. Second, it did not control for the possible health information accessed by the participants during the week between the second and the third testing. Despite statistical bootstrapping, the results cannot be generalized at population level. Finally, the study cannot answer the question whether the altered schemas persisted or faded after one week; studies of longer duration are necessary to answer this question.

## Conclusion

This study shows that highly controversial media information has large and instant effect on young people’s judgement of health issues. These effects persist, with large effect sizes, even one week after the intervention. The results fit the schema theory, and changes in judgement could mirror both, new schema formation and schema alteration. Minor fading over one week is evident. However, in contrast to pre-intervention, the modified judgements are more closely related to each other than to the pre-intervention baseline, also supporting both the shift in the direction and the persistence of this direction in judgment attributed to the novel information. The controversial nature of the information, and its adoption as personal view, highlights the dangers of the distorted, false, or unfounded information on young people’s view of various health issues. The consequences of distorted schemas are known to be very dangerous and social or governmental action in educating the vulnerable groups is urgently needed. Future research should examine the durability of the altered schemas while trying to control for life events between the testing intervals.
